# Healthcare utilization of Mexican-American Medicare beneficiaries with and without Alzheimer’s disease and related dementias

**DOI:** 10.1371/journal.pone.0227681

**Published:** 2020-01-15

**Authors:** Brian Downer, Soham Al Snih, Mukaila Raji, Lin-Na Chou, Yong-Fang Kuo, Kyriakos S. Markides, Kenneth J. Ottenbacher

**Affiliations:** 1 University of Texas Medical Branch, School of Health Professions, Division of Rehabilitation Sciences, Galveston, Texas, United States of America; 2 University of Texas Medical Branch, Sealy Center on Aging, Galveston, Texas, United States of America; 3 University of Texas Medical Branch, Internal Medicine–Geriatrics, Galveston, Texas, United States of America; 4 University of Texas Medical Branch, Office of Biostatistics, Galveston, Texas, United States of America; 5 University of Texas Medical Branch, Preventive Medicine and Population Health, Galveston, Texas, United States of America; University of Alicante, SPAIN

## Abstract

**Background:**

Older adults with Alzheimer’s disease and related dementias (ADRD) are high-risk to experience hospitalizations and emergency room (ER) admissions. Mexican-Americans have a high prevalence of ADRD, but there is limited information on the healthcare use of older Mexican-Americans with ADRD. We used data from a cohort of older Mexican-Americans that has been linked with Medicare files to investigate differences in hospitalizations, ER admissions, and physician visits according to ADRD diagnosis. We also identify sociodemographic, health, and functional characteristics that may contribute to differences in healthcare utilization between Mexican-American Medicare beneficiaries with and without an ADRD diagnosis.

**Methods and findings:**

Data came from the Hispanic Established Populations for the Epidemiological Study of the Elderly that has been linked with Medicare Master Beneficiary Summary Files, Medicare Provider Analysis and Review files, Outpatient Standard Analytic files, and Carrier files. The final analytic sample included 1048 participants. Participants were followed for two years (eight quarters) after their survey interview. Generalized estimating equations were used to estimate the probability for one or more hospitalizations, ER admissions, and physician visits at each quarter. ADRD was associated with higher odds for hospitalizations (OR = 1.65, 95%CI = 1.29–2.11) and ER admissions (OR = 1.57, 95%CI = 1.23–1.94) but not physician visits (OR = 1.23, 95%CI = 0.91–1.67). The odds for hospitalizations (OR = 1.24, 95%CI = 0.97–1.60) and ER admissions (OR = 1.27, 95%CI = 1.01–1.59) were reduced after controlling for limitations in activities of daily living and comorbidities.

**Conclusions:**

Mexican-American Medicare beneficiaries with ADRD had significantly higher odds for one or more hospitalizations and ER admissions but similar physician visits compared to beneficiaries without ADRD. Functional limitations and comorbidities contributed to the higher hospitalizations and ER admissions for older Mexican-Americans with ADRD.

## Introduction

Hispanics are a high-risk population for Alzheimer’s disease and related dementias (ADRD). The prevalence of ADRD among Hispanics aged 65 and older is nearly 17%, which is over two times higher than non-Hispanic Whites of the same age [1]. Additionally, the number of older Hispanic adults living with ADRD is projected to increase from 430 thousand in 2014 to 1 million in 2030 [2]. This dramatic increase will be due largely to population aging, but the high prevalence of important risk factors such as diabetes [3] and low education [4] will also have an important role.

The sociodemographic and health characteristics that make older Hispanics a high-risk population for ADRD also contribute to high rates of hospitalizations [5] and emergency room (ER) visits [6]. Language barriers [7] and limited access to high-quality care [8] have a role in the lower use of physician and outpatient services by Hispanics than non-Hispanic whites [9]. However, the use of outpatient services by Mexican-American Medicare beneficiaries is high and nearly 80% of beneficiaries have one or more outpatient visits every three months [10].

Older adults diagnosed with ADRD are more likely to be hospitalized [11, 12], have longer hospital stays [13], and have more visits to the ER [14] than older adults who have not been diagnosed with ADRD. The cognitive, behavioral, and functional symptoms of ADRD all contribute to the high healthcare utilization [15]. Greater functional limitations have been associated with significantly higher odds for hospitalization from diabetes, urinary tract infections, and dehydration and longer hospital stay for Medicare beneficiaries with ADRD [16].

Prior research on differences in healthcare utilization by ADRD diagnosis has used data from cohorts that are predominately non-Hispanic White. Social and cultural factors add complexity to understanding the healthcare utilization of older Hispanics who have been diagnosed with ADRD. Dementia has a strong negative stigma in Hispanic culture [17], which can perpetuate misconceptions about ADRD. A common misconception in the general population, but especially among Hispanics [18], is ADRD symptoms are a normal part of aging. Not recognizing early changes in cognitive function as a sign of ADRD may contribute to Hispanics experiencing declines in memory for a longer period of time before being diagnosed with ADRD [19] and having more severe symptoms when ADRD is diagnosed compared to non-Hispanic whites [20, 21]. Some [22], but not all [23] studies have reported that Hispanics who have been diagnosed with ADRD are less likely than non-Hispanic whites to be taking medications for ADRD symptoms. The more severe ADRD symptoms and less treatment of symptoms among older Hispanics may contribute to increased healthcare utilization in this population.

Research on differences in healthcare utilization among older Mexican-Americans with and without ADRD is limited. An analysis of 1,090 participants from the Hispanic Established Populations for the Epidemiological Study of the Elderly revealed that decedents with an ADRD diagnosis were 0.67 times less likely to be hospitalized during the last 30-days of life when compared to non-ADRD decedents [24].

The current study uses survey data from a cohort of Mexican-American adults aged 75 and older that has been linked with Medicare files. We use this unique data source to compare 2-year quarterly trends in the probability for hospitalizations, ER admissions, and physician visits according to ADRD diagnosis and identify sociodemographic, health, and functional characteristics that may contribute to differences in healthcare utilization between Mexican-American Medicare beneficiaries with and without an ADRD diagnosis. We also investigate differences according to ADRD diagnosis in the health conditions that contribute to hospitalizations. We hypothesize that Mexican-American Medicare beneficiaries with ADRD will have higher odds for hospitalizations and ER admissions than beneficiaries who have not been diagnosed with ADRD. We also hypothesize that these differences in healthcare utilization will be due to the higher number of health comorbidities and greater functional limitations among Mexican-American beneficiaries with ADRD.

## Materials and methods

### Sample population

We used data from Wave 5 (2004/05) of the Hispanic Established Populations for the Epidemiological Study of the Elderly (H-EPESE) that has been linked with Medicare claims files. The H-EPESE began in 1993/94 and included a representative sample of 3,050 participants aged 65 and older living in Texas, New Mexico, Colorado, Arizona, and California [25]. Follow-up observation waves have been completed every two to three years with the most recent wave of data collection (Wave 9) being completed in 2016. A new, representative sample of 902 participants aged 75 and older was added to the sample at Wave 5 (2004/05).

A detailed description of the procedure for linking the H-EPESE survey with Medicare files has been provided [10]. Waves 4 (1999/2000) through 8 (2012/13) of the H-EPESE have been linked with Medicare Master Beneficiary Summary Files, Medicare Provider Analysis and Review (MedPAR) files, Outpatient Standard Analytic files (OUTSAF), and Carrier files for 1999 to 2012. We used survey data from Wave 5 for information on participants’ self-reported demographic characteristics, activities of daily living, and cognitive functioning. Wave 5 was selected so that participants who were recruited to the H-EPESE in 2004/05 could be included in the analysis.

**Fig 1:**
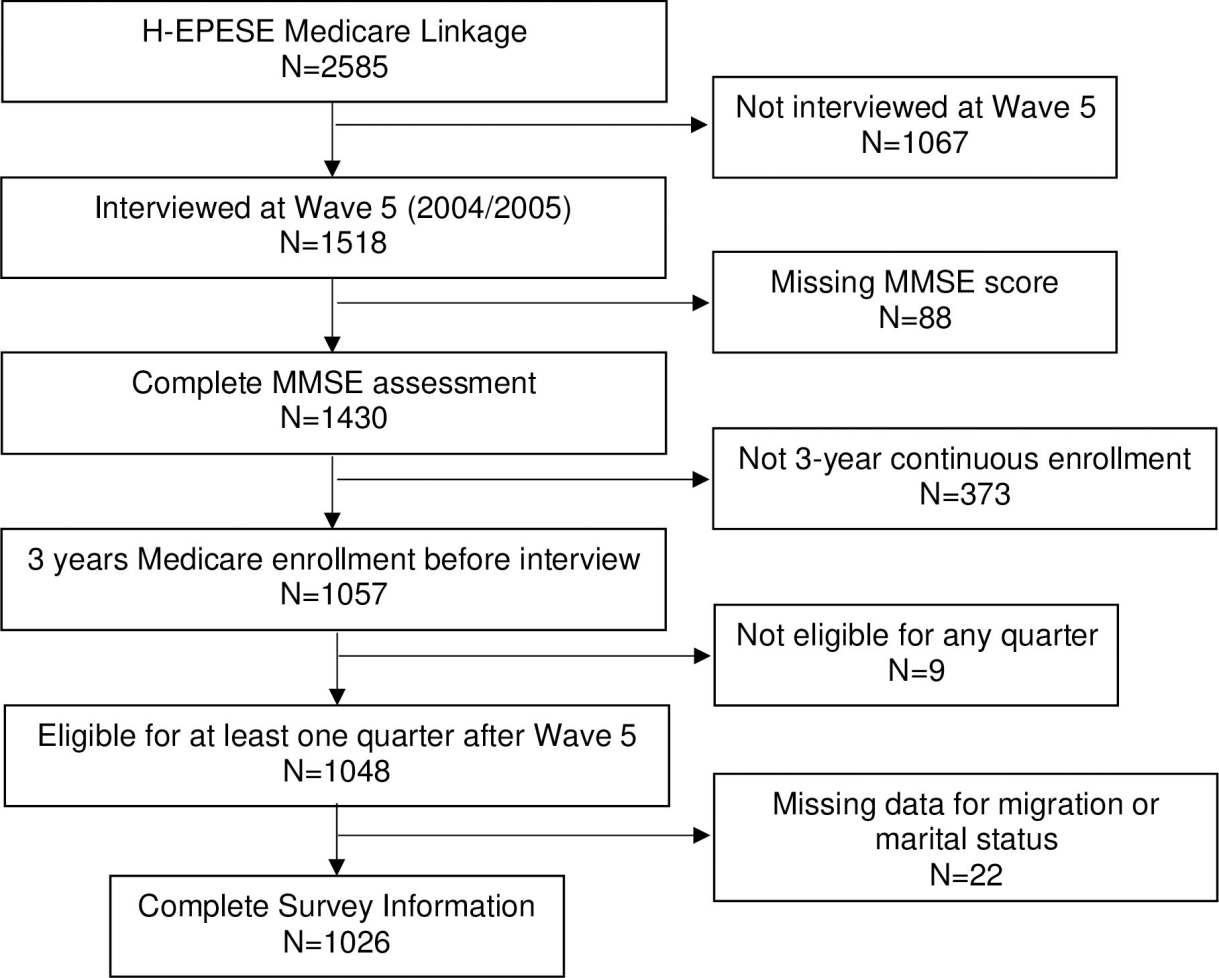
Selection of final analytic sample.

The selection of the final analytic sample is shown in Fig 1. Data for 2,585 participants were linked with Medicare claims files of which 1,518 participants were interviewed at Wave 5. We excluded participants who were missing information for cognitive functioning (n = 88), were not continuously enrolled in Medicare fee-for-service Part A and Part B coverage for the three-years prior to the Wave 5 interview (n = 373), and who were not Medicare Part A and B eligible for at least one quarter in the two-year period (eight quarters) after the Wave 5 interview (n = 9). This sample of 1,048 participants was used to evaluate the descriptive characteristics according to ADRD status and describe unadjusted quarterly trends in healthcare utilization by ADRD status. The multivariable regression analyses used an analytic sample of 1,026 participants because 22 participants did not provide information for migration history and marital status.

We evaluated participants’ eligibility at each quarter for a total of eight quarters after a participant’s Wave 5 interview until the quarter before death. Participants had to have Medicare fee-for-service coverage for three months to be eligible in a quarter. The Wave 5 interviews were completed between June, 2004 and December, 2005. The start of year 1 quarter 1 was the first full month after each participant’s Wave 5 interview.

### Classification of Alzheimer’s disease and related dementias

ADRD status was determined using the Chronic Conditions Segment of the Master Beneficiary Summary File. The Chronic Conditions Segment is a list of twenty-seven conditions which includes a diagnosis of Alzheimer’s disease and related disorders or senile dementia. The flag variable for ADRD is created using an algorithm in which at least one out of 24 ICD-9 codes must be present in one or more inpatient, skilled nursing, home health, Part B institutional, or carrier file claims. The Chronic Conditions Segment also includes the date in which a diagnosis for ADRD first occurred. We used this information to identify participants who had an ADRD diagnosis prior to the date of their Wave 5 interview.

### Healthcare utilization

The outcome measures were acute hospitalizations, ER admissions, and physician visits. We identified participants who had one or more hospitalization at each quarter by using the Medicare Provider Analysis and Review (MedPAR) file and selecting acute hospital and critical access hospital stays according to provider number. We used the admission date to determine which quarter the hospitalization occurred. ER admissions were identified as an ER revenue code (0450, 0451, 0452, 0456, 0459, 0981) in the Outpatient Standard Analytic Files (OUTSAF) or if an ER charge amount of more than zero dollars was identified in the MedPAR file. Participants were dichotomized according to having one or more ER admissions per quarter. Physician visits were identified according to current procedural terminology codes for new (99201–99205) and established (99211–99215) patient office or other physician services in the OUTSAF and Carrier files. Participants were dichotomized according to having one or more physician visits per quarter.

An additional analysis was done to investigate potential differences according to ADRD status for medical conditions that contributed to hospitalizations. We identified common medical conditions by grouping the primary hospital discharge diagnoses into 25 major diagnostic categories. We calculated the percentage of participants who had a discharge diagnosis for each major diagnostic category among the 460 participants who had one or more hospitalizations during all eligible quarters. The denominators for all calculations was 109 for participants with ADRD and 351 participants with no ADRD diagnosis. The numerator was the number of participants with a hospitalization for each major diagnostic category. This accounted for participants who had multiple hospitalizations.

### Covariates

Covariates from the Wave 5 survey included age (75–79, 80–84, and 85+), gender, education (0, 1–4, 5+ years), nativity, marital status, completing the survey in English or Spanish, marital status (married, not married), cognitive impairment, and activities of daily living (ADLs). We also included a variable that indicated if a participant was recruited to the H-EPESE in 1993/94 or 2004/05. Nativity status was categorized as being born in the U.S. or the age of migration to the U.S. for participants who were born in Mexico (0–19, 20+ years old). Participants were dichotomized according to needing help from another person for one or more ADLs (walking, bathing, grooming, dressing, eating, toileting, and moving from a bed to a chair). Cognitive functioning was measured using the Mini Mental Status Exam (MMSE) [26]. Participants who scored less than 21 points on the MMSE were classified as cognitively impaired [27].

We also controlled for mortality and number of health comorbidities according to the Charlson Comorbidity Index (CCI). The H-EPESE has been linked with the National Death Index. Mortality status from the National Death Index was used to identify participants who died within 2-years of their Wave 5 interview, those who died between 2 and 3 years of their Wave 5 interview, and participants who were alive for three years after their wave 5 interview. The CCI is a list of 17 comorbidities [28]. We used a list of 16 conditions because dementia is included in the CCI. We used inpatient and physician files for the one-year prior to the Wave 5 interview to calculate the CCI. Participants were categorized as having 0, 1–2, 3–5, or 5+ comorbidities.

### Statistical analysis

Generalized estimating equations were used to determine quarterly trends in the probability of having one or more hospitalizations, ER admissions, and physician visits according to ADRD status. We report adjusted odds ratios and predicted probabilities that were estimated using marginal standardization [29]. Marginal standardization is the weighted average of the predicted probabilities for each value of the covariates. Three models were created. Model 1 controlled for time (quarter), cohort, age at Wave 5 interview, gender, nativity and age of migration, education, marital status, language, and mortality status. Mortality was included in the base model because of the increased risk for mortality associated with ADRD and increasing healthcare utilization prior to death. Model 2 controlled for the covariates in model 1 plus cognitive impairment. Model 3 controlled for the covariates in model 2 plus number of comorbidities and having one or more ADL limitations. The three models were used to determine if differences in healthcare utilization by ADRD status were mediated by cognitive impairment, chronic conditions, and ADL limitations. Analyses were done using SAS 9.4 (SAS Inc., Cary, NC) and Stata 15.1 (StataCorp, College Station, TX).

### Ethics statement

The current study involved secondary analysis of existing H-EPESE data files that have been de-identified. No new primary data from human subjects were collected. Accordingly, the institutional review board (IRB) at the University of Texas Medical Branch approved the current study as falling under Exemption 4 of the National Institute of Health guidelines (IRB # 16–0014).

All aspects of the H-EPESE protocol have been approved by the University of Texas Medical Branch IRB (IRB # 92–85). All interviews were performed in subjects’ homes by trained, bilingual interviewers employed by Harris Interactive Inc. An interviewer obtained written or oral consent from a subject prior to the start of the interview. Subjects’ participation was completely voluntary and a subject could refuse to participate at any time. The H-EPESE protocol approved by the University of Texas Medical Branch IRB did not include an assessment to determine a subject’s capacity to provide written or oral consent. However, a spouse, adult child, or other close family member was interviewed as a proxy if a subject was unable to complete or attempt the interview because of illness, cognitive impairment, or any other reason. All H-EPESE interviewers received individualized training from members of the research team including an ethicist and senior investigators with IRB experience.

## Results

The demographic and health characteristics of the final sample according to ADRD status are shown in Table 1. A total of 193 (18.4%) participants had a diagnosis of ADRD. These participants were significantly older, completed fewer years of education, and were more likely to need help with one or more ADLs, to be cognitively impaired, to have five or more comorbidities, and to be deceased within three years of the Wave 5 interview.

**Table 1:** Demographic and health characteristics of H-EPESE subjects according to ADRD status in 2004/05 (n = 1048).

Variable	ADRD N = 193	Non-ADRD N = 855	p-value
Cohort			0.05
1993/94	146 (75.6)	586 (68.5)	
2004/05	47 (24.4)	269 (31.5)	
Age at interview			
Mean (SD), years	83.6 (5.66)	81.4 (4.73)	<0.01
75–79	55 (28.5)	366 (42.8)	<0.01
80–84	62 (32.1)	290 (33.9)	
85+	76 (39.4)	199 (23.3)	
Gender			0.56
Male	73 (37.8)	343 (40.1)	
Female	120 (62.2)	512 (59.9)	
Age of migration*			0.29
US Born	106 (56.4)	504 (59.9)	
0–19	22 (11.7)	69 (8.2)	
20+	60 (31.9)	268 (31.9)	
Education			
Mean (SD), years	3.8 (3.59)	4.9 (3.95)	<0.01
No formal education	47 (24.4)	142 (16.6)	<0.01
Elementary school	94 (48.7)	376 (44.0)	
Middle-school or higher	52 (26.9)	337 (39.4)	
Marital status*			0.78
Married	80 (41.7)	365 (42.8)	
Not married	112 (58.3)	488 (57.2)	
Language at interview			0.26
English	28 (14.5)	153 (17.9)	
Spanish	165 (85.5)	702 (82.1)	
ADL			<0.01
Not need help	63 (32.6)	583 (68.2)	
Need ≥ 1 help	130 (67.4)	272 (31.8)	
Cognitively impaired			<0.01
No	63 (32.6)	552 (64.6)	
Yes	130 (67.4)	303 (35.4)	
Charlson comorbidity			
Number of condition	3.2 (2.20)	1.9 (1.84)	<0.01
0 comorbidity	20 (10.4)	239 (28.0)	<0.01
1–2 comorbidities	64 (33.2)	326 (38.1)	
3–4 comorbidities	59 (30.6)	198 (23.2)	
≥ 5 comorbidities	50 (25.9)	92 (10.8)	
Mortality			<0.01
Alive	121 (62.7)	681 (79.6)	
Deceased within 2 years	47 (24.4)	99 (11.6)	
Deceased during 2–3 years	25 (13.0)	75 (8.8)	

*Twenty-two participants did not report information for marital status or migration at the wave 5 survey interview.

**Fig 2:**
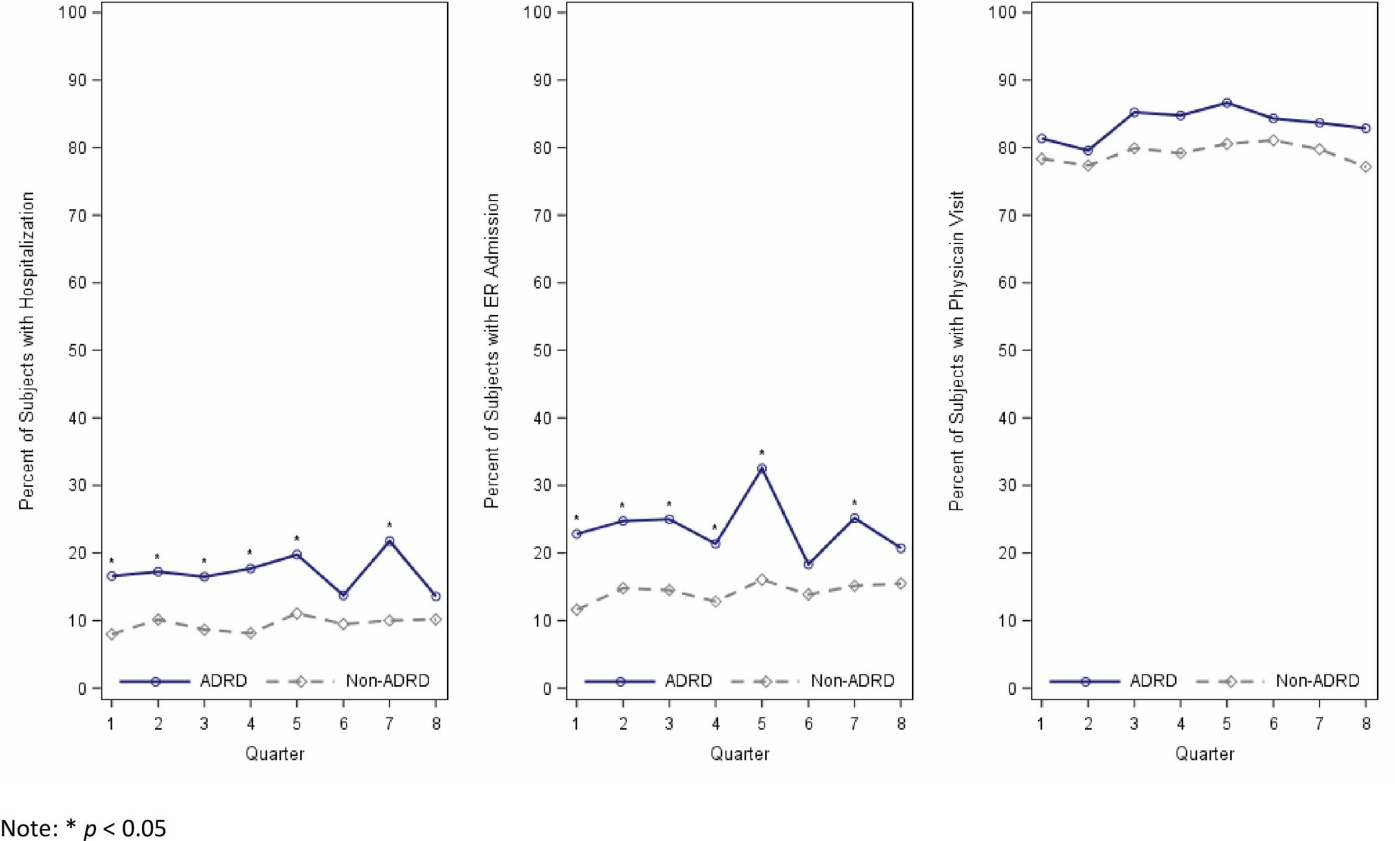
Observed percentage of participants with one or more hospitalizations, ER admissions, and physician visits per quarter according to ADRD status.

The percentage of participants with an ADRD diagnosis who had one or more hospitalizations, ER admissions, and physician visits per quarter ranged from 13.57% to 21.77%, 18.30% to 32.48%, and 79.57% to 86.62%, respectively (Fig 2). The percentage of participants who had not been diagnosed with ADRD who had one or more hospitalizations, ER admissions, and physician visits per quarter ranged from 7.96% to 11.04%, 11.59% to 16.03%, and 77.17% to 81.07%, respectively. With the exception of quarter 6 and quarter 8, the percentage of participants with ADRD who had one or more hospitalizations and ER admissions were significantly greater at every quarter than participants who did not have ADRD (Fig 2). There were no significant differences by ADRD status at any quarter for the percentage of participants who had one or more physician visits.

Participants with ADRD had 1.65 (95% CI = 1.29–2.11) higher odds for one or more hospitalizations and 1.57 (95% CI = 1.26–1.95) higher odds for one or more ER admissions when controlling for demographic characteristics and mortality (Table 2). The higher odds for one or more hospitalizations (OR = 1.69, 95% CI = 1.29–2.20) and one or more ER admissions (OR = 1.55, 95% CI = 1.23–1.94) for participants with ADRD remained statistically significant after controlling for cognitive impairment. The increased odds for one or more hospitalizations (OR = 1.24, 95% CI = 0.97–1.60) and one or more ER admissions (OR = 1.27, 95% CI = 1.01–1.59) for participants with ADRD were reduced after controlling for number of comorbidities and limitations in one or more ADLs. No statistically significant differences in the odds for one or more physician visits according to ADRD status were detected in any of the models.

**Table 2:** Predicted probability and adjusted odds ratios (aOR) for hospitalizations, ER admissions, and physician visits by ADRD status (N = 1026).

	Hospitalization		ER Admission		Physician Visit	
	% (SE)	aOR (95% CI)	% (SE)	aOR (95% CI)	% (SE)	aOR (95% CI)
Model 1						
Non-ADRD	9.8 (0.48)	1.00	14.6 (0.60)	1.00	78.9 (1.00)	1.00
ADRD	14.8 (1.19)	1.65 (1.29–2.11)*	20.8 (1.45)	1.57 (1.26–1.95)*	82.1 (2.06)	1.23 (0.91–1.67)
Model 2						
Non-ADRD	9.7 (0.48)	1.00	14.6 (0.60)	1.00	78.7 (1.01)	1.00
ADRD	15.0 (1.24)	1.69 (1.29–2.20)*	20.6 (1.48)	1.55 (1.23–1.94)*	82.8 (2.02)	1.31 (0.96–1.79)
Model 3						
Non-ADRD	10.3 (0.47)	1.00	15.1 (0.59)	1.00	79.6 (0.91)	1.00
ADRD	12.2 (1.01)	1.24 (0.97–1.60)	18.2 (1.31)	1.27 (1.01–1.59)*	78.5 (2.28)	0.93 (0.67–1.30)

*p < 0.05

standard error (SE); Percentages calculated using marginal standardization

Model 1: Time (quarter), cohort, age, gender, age of migration, education, marital status, language, and mortality

Model 2: model 1 covariates plus cognitive impairment

Model 3: model 2 covariates plus comorbidities and 1+ ADL limitations

Table 3 presents the adjusted odds ratios and predicted probabilities for all variables in the fully adjusted model. The adjusted odds for hospitalizations and ER admissions increased over time and were significantly higher for participants recruited into the H-EPESE in 2004/05 compared to those recruited into the Study in 1993/94. Participants age 80–84 and 85 or older had 1.24 (95% CI = 1.01–1.52) and 1.41 (95% CI = 1.13–1.75) higher odds for ER admissions, respectively compared to participants aged 75–79. Spanish language was associated with significantly higher odds for hospitalizations (OR = 1.53, 95% CI = 1.15–2.05) and physician visits (OR = 1.37, 1.02–1.84). Mortality and number of health comorbidities were associated with higher odds for hospitalizations and ER admissions. A greater number of health comorbidities was also associated with significantly higher adjusted odds for physician visits. Women had 1.59 higher odds (95% CI = 1.21–2.08) to have a physician visit than men. Having one or more ADL limitations was associated with higher odds for hospitalizations (OR = 1.40, 95% CI = 1.13–1.73) whereas cognitive impairment was associated with lower odds for physician visits (OR = 0.73, 95% CI = 0.56–0.95).

**Table 3:** Predicted probabilities and adjusted odds ratios for hospitalizations, ER admissions, and physician visits (N = 1063).

	Hospitalization		ER Admission		Physician Visit	
Characteristics	%(SE)	aOR (95% CI)	%(SE)	aOR (95% CI)	%(SE)	aOR (95% CI)
Time, quarter		1.08 (1.04–1.12)*		1.06 (1.03–1.10)*		1.01 (0.99–1.03)
ADRD diagnosis						
No	10.3 (0.47)	1.00	15.1 (0.59)	1.00	79.6 (0.91)	1.00
Yes	12.2 (1.01)	1.24 (0.97–1.60)	18.2 (1.31)	1.27 (1.01–1.59)*	78.5 (2.28)	0.93 (0.67–1.30)
Cohort						
1993/94	10.1 (0.49)	1.00	14.9 (0.62)	1.00	79.2 (1.00)	1.00
2004/05	12.1 (0.79)	1.26 (1.01–1.55)*	17.6 (1.00)	1.24 (1.04–1.49)*	80.1 (1.53)	1.06 (0.83–1.35)
Age at interview						
75–79	9.7 (0.66)	1.00	13.7 (0.81)	1.00	80.2 (1.31)	1.00
80–84	11.6 (0.75)	1.24 (0.97–1.58)	16.2 (0.93)	1.24 (1.01–1.52)*	80.9 (1.37)	1.05 (0.81–1.37)
85+	11.0 (0.81)	1.16 (0.93–1.45)	17.9 (1.09)	1.41 (1.13–1.75)*	76.3 (1.84)	0.78 (0.58–1.05)
Gender						
Male	11.2 (0.73)	1.00	14.8 (0.87)	1.00	75.3 (1.53)	1.00
Female	10.4 (0.55)	0.91 (0.73–1.14)	16.3 (0.72)	1.13 (0.93–1.37)	82.3 (1.08)	1.59 (1.21–2.08)*
Age of migration, year						
US Born	10.9 (0.56)	1.00	16.1 (0.71)	1.00	78.6 (1.11)	1.00
0–19	12.5 (1.48)	1.18 (0.86–1.63)	17.2 (1.78)	1.10 (0.82–1.47)	81.5 (2.70)	1.22 (0.84–1.79)
20+	9.8 (0.72)	0.88 (0.70–1.10)	14.7 (0.92)	0.89 (0.73–1.09)	80.6 (1.53)	1.15 (0.88–1.51)
Education						
No formal education	10.6 (0.94)	1.00	17.5 (1.28)	1.00	80.0 (2.09)	1.00
Elementary school	10.4 (0.62)	0.98 (0.75–1.28)	15.4 (0.79)	0.85 (0.68–1.08)	80.5 (1.25)	1.03 (0.75–1.42)
Middle-school or higher	11.2 (0.77)	1.07 (0.79–1.43)	15.1 (0.92)	0.83 (0.64–1.07)	78.0 (1.47)	0.88 (0.63–1.22)
Marital status						
Married	10.5 (0.68)	1.00	16.0 (0.89)	1.00	79.2 (1.36)	1.00
Not married	10.9 (0.58)	1.05 (0.85–1.30)	15.5 (0.71)	0.96 (0.80–1.17)	79.6 (1.18)	1.03 (0.78–1.35)
Language						
English	7.9 (0.91)	1.00	14.3 (1.26)	1.00	75.4 (2.21)	1.00
Spanish	11.3 (0.47)	1.53 (1.15–2.05)*	16.0 (0.59)	1.15 (0.92–1.45)	80.3 (0.91)	1.37 (1.02–1.84)*
ADL Limitation						
Not need help	9.4 (0.56)	1.00	15.3 (0.73)	1.00	78.2 (1.10)	1.00
Need ≥ 1 help	12.4 (0.73)	1.40 (1.13–1.73)*	16.3 (0.87)	1.08 (0.90–1.30)	81.9 (1.49)	1.28 (0.99–1.66)
Cognitive function						
Normal (MMSE≥21)	11.5 (0.63)	1.00	15.5 (0.76)	1.00	81.3 (1.08)	1.00
Impairment (MMSE<21)	9.9 (0.63)	0.83 (0.65–1.06)	15.9 (0.86)	1.03 (0.85–1.26)	76.5 (1.54)	0.73 (0.56–0.95)*
Charlson comorbidity						
0 comorbidity	5.2 (0.66)	1.00	9.2 (0.90)	1.00	63.9 (2.08)	1.00
1–2 comorbidities	9.5 (0.68)	1.96 (1.45–2.64)*	14.6 (0.85)	1.71 (1.34–2.19)*	81.8 (1.34)	2.62 (2.01–3.41)*
3–4 comorbidities	12.3 (0.91)	2.64 (1.95–3.58)*	18.2 (1.14)	2.25 (1.73–2.92)*	86.1 (1.49)	3.64 (2.62–5.06)*
≥ 5 comorbidities	19.4 (1.52)	4.72 (3.38–6.61)*	25.2 (1.82)	3.48 (2.56–4.74)*	92.0 (1.66)	6.81 (4.25–10.90)*
Mortality						
Alive	8.4 (0.44)	1.00	13.7 (0.58)	1.00	80.1 (0.92)	1.00
Dead within 2 years	28.2 (2.18)	4.68 (3.62–6.06)*	30.9 (2.31)	2.97 (2.31–3.82)*	78.5 (2.79)	0.90 (0.61–1.33)
Dead during 2–3 years	13.2 (1.39)	1.71 (1.26–2.32)*	18.3 (1.71)	1.43 (1.07–1.91)*	74.9 (2.89)	0.72 (0.51–1.02)

The three most common major diagnostic categories among the 460 participants who had one or more hospitalizations were for conditions in the circulatory system, respiratory system, and nervous system (Table 4). There were statistically significant differences according to ADRD diagnosis for conditions of the respiratory system, nervous system, and the endocrine, nutritional, and metabolic system. Thirty-five (32.11%) participants diagnosed with ADRD were discharged for respiratory system conditions compared to 75 (21.37%) participants without ADRD. Approximately 18% of beneficiaries with ADRD were discharged for conditions of the nervous system compared to 10.54% of beneficiaries without a diagnosis of ADRD. Finally, 17.43% of beneficiaries with ADRD were discharged for endocrine, nutritional and metabolic system conditions compared to 10.26% of beneficiaries with no ADRD diagnosis.

**Table 4:** The eight most frequent major diagnosis categories for hospitalized subjects according to ADRD status (N = 460).

Major Diagnosis Category	ADRD N = 109	Non-ADRD N = 351	p-value
Circulatory System	41 (37.61%)	121 (34.47%)	0.55
Respiratory System	35 (32.11%)	75 (21.37%)	0.02*
Nervous System	20 (18.35%)	37 (10.54%)	0.03*
Musculoskeletal System and Connective Tissue	19 (17.43%)	63 (17.95%)	0.90
Endocrine, Nutritional and Metabolic System	19 (17.43%)	36 (10.26%)	0.04*
Digestive System	18 (16.51%)	69 (19.66%)	0.46
Kidney and Urinary Tract	18 (16.51%)	47 (13.39%)	0.41
Infectious and Parasitic DDs	13 (11.93%)	30 (8.55%)	0.29

*p < 0.05

## Discussion

This analysis investigated quarterly trends in hospitalizations, ER admissions, and physician visits to determine if there are differences between older Mexican-American Medicare Beneficiaries with and without a diagnosis of ADRD. The initial analysis showed that beneficiaries with a diagnosis of ADRD had significantly higher odds to have one or more hospitalizations and one or more ER admissions per quarter than beneficiaries without a diagnosis of ADRD. Beneficiaries with ADRD were more likely to have one or more physician visits than those with no ADRD diagnosis, but this difference was not statistically significant.

Our findings are consistent with reports of greater hospitalizations and ER admissions among older adults with ADRD in the general Medicare population [11, 12]. In our fully adjusted models, the predicted probabilities for one or more hospitalizations and ER admissions at each quarter were 12.2% and 18.2%, respectively. Prior studies have mostly analyzed differences in hospitalization or ER admissions rates over a 6-month [30, 31] or 1-year [32–37] period whereas we assessed quarterly (i.e. 3-month) rates. This makes it difficult to compare our findings to earlier studies. An analysis of the Washington Heights-Inwood Columbia Aging Project, which is a racially and ethnically diverse cohort, reported that the adjusted quarterly rate for one or more inpatient hospitalization was 14.2%.

We found that Mexican-American beneficiaries with an ADRD diagnosis were more likely to be cognitively impaired, to have limitations in one or more ADLs, and had more health comorbidities than beneficiaries who did not have an ADRD diagnosis. The associations between ADRD and healthcare utilization did not substantially change after controlling for cognitive impairment. However, ADRD was no longer associated with significantly higher odds for one or more hospitalizations after controlling for health comorbidities and functional limitations. Additionally, the adjusted odds ratio for one or more ER admissions was nearly twenty percent lower in the fully adjusted model compared to when controlling for baseline characteristics and cognitive impairment (Model 2). Functional limitations and health conditions also contribute to higher healthcare costs for older adults with ADRD [38] whereas evidence is mixed on if cognitive impairment impacts the healthcare cost and utilization of older adults with ADRD [15, 16].

Older adults often lose function in ADLs during a hospital stay [39] and can have difficulty returning back to their pre-hospitalization level of function [40]. ADRD is associated with less improvement in ADL function after a hospitalization [41], which may increase the risk for future hospitalizations [42]. Over half of older adults receive post-acute care at home or in a skilled nursing facility [43]. Post-acute care can improve the ADL function of older adults with ADRD [44] and cognitive impairment [45]. However, being discharged to skilled nursing or inpatient rehabilitation settings that are meant to provide intense rehabilitation may not be appropriate for all older adults with ADRD [46].

Our findings add to the growing evidence that having a greater number of health conditions is an important contributing factor to the high healthcare utilization of older adults with ADRD [11]. Chronic conditions such as diabetes, atherosclerosis, and hypertension are common among older adults with ADRD [47]. The symptoms of ADRD can complicate the treatment and management of chronic health conditions [48]. Our results suggest this may lead to increased hospitalizations, ER admissions, and more frequent engagement with outpatient services for older Mexican-Americans.

The observed percentage of beneficiaries who had one or more physician visits was slightly but not significantly higher for those with an ADRD diagnosis. In each quarter, over 80% of participants with ADRD and 78% of participants without ADRD visited a physician one or more times each quarter. This is lower than what has been reported in other studies [49]. We did observe that cognitive impairment was associated with significantly lower odds for having visited a physician. Lower use of outpatient services has been observed for older adults with severe ADRD [15], which has been attributed to desires of the patient and family for less aggressive medical care [50]. The prevalence of cognitive impairment in our sample was high and 35% of beneficiaries who did not have an ADRD diagnosis scored lower than 21 on the MMSE at the time of their interview. Continued research is needed to identify factors that contribute to the lower use of physician services for older Mexican-American beneficiaries who are cognitively impaired.

This analysis revealed that older Mexican-American beneficiaries who completed their survey interview in Spanish had significantly higher odds to visit a physician than English speakers. This finding contrasts with evidence that better English proficiency is associated greater likelihood to visit a physician [51] and that language discrimination during previous healthcare encounters contributes to delays in using healthcare services in the future [52]. Addressing discordance between patients and physicians on race, ethnicity, and language may be effective in reducing disparities in patient satisfaction [53]. However, there is little evidence that patient-physician concordance has positive effects on healthcare outcomes [54].

We explored differences in the health conditions that contributed to hospitalizations according to ADRD status by identifying the eight most frequent major diagnostic categories at hospital discharge. A significantly greater percentage of participants with ADRD were hospitalized for conditions in the respiratory system and for conditions in the endocrine, nutritional and metabolic system. Thirty-five percent of participants with ADRD were hospitalized for a respiratory system condition compared to 21.8% for participants without an ADRD diagnosis. The higher frequency of hospitalizations for conditions in these two major diagnostic categories is supported by evidence that older adults with ADRD are more likely than older adults without ADRD to be admitted to the hospital for pneumonia[55], other respiratory infections [56], and complications from diabetes [12]. The relatively small sample size of the H-EPESE prevented us from determining in greater detail the specific health conditions within these categories that contributed to hospitalizations.

### Strengths and limitations

A strength of this analysis is that we used Medicare claims files that have been linked with survey data from the H-EPESE. This allowed use to identify characteristics associated with healthcare utilization that are not included in Medicare claims files, such as ADL limitations, cognitive impairment, and language. This study also has limitations. First, Medicare claims data can have poor sensitivity for detecting ADRD [57]. An analysis by Zhu et al. [58] reported that Hispanics who had a clinical diagnosis of dementia that was made by an expert panel were more likely than non-Hispanic whites to be incorrectly classified as not having ADRD according to Medicare claims files. The possibility of participants’ ADRD status being misclassified is a concern given that 35% of participants in our sample who did not have an ADRD diagnosis were classified as cognitively impaired according to the MMSE. Conversely, 32.6% of participants with an ADRD diagnosis were classified as cognitively intact at the Wave 5 interview. This may be due in part to cultural biases of the MMS that reduce its accuracy in screening for dementia in Hispanic populations [59].

A second limitation is our findings are not generalizable to other Hispanic populations in the U.S. because the H-EPESE only includes participants of Mexican origin. Mexican-Americans are the largest Hispanic population in the U.S. [60], but the majority of Hispanics in the Northeast and Southeast U.S. are of Cuban, Puerto Rican, and Dominican descent [61]. Caribbean Hispanics are generally more highly educated and have fewer health conditions than Mexican-Americans [62]. This may contribute to differences in healthcare utilization among Hispanic sub-populations.

### Conclusions

In summary, greater functional limitations and health comorbidities contribute to the higher hospitalizations and ER admissions for Mexican-American Medicare beneficiaries with ADRD than those with no ADRD diagnosis. We did not detect significant differences in physician visits by ADRD status. Our findings can contribute to improving public health by identifying additional factors that contribute to increased hospitalizations and ER admissions by older Mexican-Americans with ADRD. Our findings are also relevant to national policy makers who are responsible for ensuring that communities have sufficient healthcare resources that can meet the needs of a growing Hispanic population that is rapidly aging.
